# Berry skin development in Norton grape: Distinct patterns of transcriptional regulation and flavonoid biosynthesis

**DOI:** 10.1186/1471-2229-11-7

**Published:** 2011-01-10

**Authors:** Mohammad B Ali, Susanne Howard, Shangwu Chen, Yechun Wang, Oliver Yu, Laszlo G Kovacs, Wenping Qiu

**Affiliations:** 1 Center for Grapevine Biotechnology, William H. Darr School of Agriculture, Missouri State University, Mountain Grove, MO 65711, USA; 2 The Donald Danforth Plant Science Center, St. Louis, MO 63132, USA; 3 College of Food Sciences and Nutritional Engineering, China Agricultural University, Beijing 100083, PR China; 4 Department of Plant and Soil Sciences, University of Kentucky, Lexington, KY 40546, USA

## Abstract

**Background:**

The complex and dynamic changes during grape berry development have been studied in *Vitis vinifera*, but little is known about these processes in other *Vitis *species. The grape variety 'Norton', with a major portion of its genome derived from *Vitis aestivalis*, maintains high levels of malic acid and phenolic acids in the ripening berries in comparison with *V. vinifera *varieties such as Cabernet Sauvignon. Furthermore, Norton berries develop a remarkably high level of resistance to most fungal pathogens while Cabernet Sauvignon berries remain susceptible to those pathogens. The distinct characteristics of Norton and Cabernet Sauvignon merit a comprehensive analysis of transcriptional regulation and metabolite pathways.

**Results:**

A microarray study was conducted on transcriptome changes of Norton berry skin during the period of 37 to 127 days after bloom, which represents berry developmental phases from herbaceous growth to full ripeness. Samples of six berry developmental stages were collected. Analysis of the microarray data revealed that a total of 3,352 probe sets exhibited significant differences at transcript levels, with two-fold changes between at least two developmental stages. Expression profiles of defense-related genes showed a dynamic modulation of nucleotide-binding site-leucine-rich repeat (NBS-LRR) resistance genes and pathogenesis-related (PR) genes during berry development. Transcript levels of *PR-1 *in Norton berry skin clearly increased during the ripening phase. As in other grapevines, genes of the phenylpropanoid pathway were up-regulated in Norton as the berry developed. The most noticeable was the steady increase of transcript levels of stilbene synthase genes. Transcriptional patterns of six MYB transcription factors and eleven structural genes of the flavonoid pathway and profiles of anthocyanins and proanthocyanidins (PAs) during berry skin development were analyzed comparatively in Norton and Cabernet Sauvignon. Transcriptional patterns of *MYB5A *and *MYB5B *were similar during berry development between the two varieties, but those of *MYBPA1 *and *MYBPA2 *were strikingly different, demonstrating that the general flavonoid pathways are regulated under different MYB factors. The data showed that there were higher transcript levels of the genes encoding flavonoid-3'-O-hydroxylase (*F3*'*H*), flavonoid-3',5'-hydroxylase (*F3'5'H*), leucoanthocyanidin dioxygenase *(LDOX)*, UDP-glucose:flavonoid 3'-O-glucosyltransferase (*UFGT*), anthocyanidin reductase (*ANR*), leucoanthocyanidin reductase (*LAR*) *1 *and *LAR2 *in berry skin of Norton than in those of Cabernet Sauvignon. It was also found that the total amount of anthocyanins was markedly higher in Norton than in Cabernet Sauvignon berry skin at harvest, and five anthocyanin derivatives and three PA compounds exhibited distinctive accumulation patterns in Norton berry skin.

**Conclusions:**

This study provides an overview of the transcriptome changes and the flavonoid profiles in the berry skin of Norton, an important North American wine grape, during berry development. The steady increase of transcripts of *PR-1 *and stilbene synthase genes likely contributes to the developmentally regulated resistance during ripening of Norton berries. More studies are required to address the precise role of each stilbene synthase gene in berry development and disease resistance. Transcriptional regulation of *MYBA1*, *MYBA2*, *MYB5A *and *MYBPA1 *as well as expression levels of their putative targets *F3*'*H, F3*'*5*'*H, LDOX*, *UFGT*, *ANR, LAR1*, and *LAR2 *are highly correlated with the characteristic anthocyanin and PA profiles in Norton berry skin. These results reveal a unique pattern of the regulation of transcription and biosynthesis pathways underlying the viticultural and enological characteristics of Norton grape, and yield new insights into the understanding of the flavonoid pathway in non-vinifera grape varieties.

## Background

Berry development in grapes is a complex process of physiological and biochemical changes [1]. It is initiated by hormonal signals generated after pollination [2]. The nature and origin of the hormonal signals that influence the complex processes of berry development have not been fully understood, but abscisic acid, brassinosteroids and ethylene have been implicated in these processes [3,4]. Although ethylene is present at the beginning of ripening, it does not show a rapid increase in concentration, and no burst of respiration occurs in grape berries [5]. Thus, grapes are non-climacteric fruits.

The berry development of grape follows a double-sigmoid pattern that is characterized by two growth phases interrupted by a lag phase (véraison) which marks the transition from herbaceous development to ripening [6]. High-throughput profiling of transcripts by using the first generation Affymetrix Vitis GeneChip has provided a comprehensive picture of gene regulation that depicts the complex biochemical pathways during berry development of *V. vinifera *grapevines [7,8]. The transcriptome analysis has also identified distinct transcriptional patterns and tissue-specific genes in seed, skin and pulp of grape berry [9]. The results of these studies have offered the insights into how key regulatory circuits orchestrate berry development and influence unique berry characteristics in *V. vinifera *varieties.

The skin of grape berries serves as a physical and biochemical barrier that protects ripening berries from being attacked by pathogens. During the first growth phase, the skin accumulates high levels of proanthocyanidins (PAs). The astringent properties of PAs may play a role in repelling herbivores from consuming berries before seeds are mature, and also in the protection of plants against fungal pathogens [10]. At véraison, the skin begins to accumulate anthocyanins which are the predominant pigments of grape berries. The dark color is believed to attract herbivorous animals to promote the dissemination of seeds into new territories. Supporting this proposition is the fact that the skin color of wild *Vitis *species berries is black. In addition to PAs and anthocyanins, the skin also accumulates flavan-3-ol monomers, although the majority of flavan-3-ols are synthesized in the grape seed [11]. The endo- and mesocarp of the berry contain large quantities of acids, primarily malic and tartaric acids, during the first growth phase, and sugars during the second growth phase of berry development [1,2].

Prior to maturity, the skin's resistance against pathogens increases in order to protect the ripening grape berries [12-14]. The high levels of flavonoid compounds in the skin are thought to contribute to the enhanced disease resistance of mature berries. It was discovered that many highly expressed genes in the skin of Cabernet Sauvignon are associated with pathogen resistance and flavonoid biosynthesis [9]. The transcriptional profiles of skin-specific genes, which were also corroborated by proteomics analysis, indicated that a set of enzymes in the anthocyanin biosynthesis pathway were significantly over-expressed in the skin of fully ripe berries [15]. A set of pathogenesis-related (*PR*) genes, such as *PR-1, PR-2, PR-3, PR-4 *and *PR-5*, all increased in the ripening berry of Cabernet Sauvignon, with *PR-3 *and *PR-5 *having the most dramatic increase [7,16]. During véraison, the berry experiences a burst of reactive oxygen species (ROS) and a surge in the expression of genes that encode enzymes involved in the generation of antioxidants [8]. Generation of ROS is closely associated with cell death and plant defense responses [17]. The timing of accumulation of these defense-related proteins is synchronized with the initiation of the ripening berry's ability to prevent infection by pathogens [18]. There is experimental evidence that the increased expression of defense-related genes forms a protective layer in the berry skin against pathogens [19,15]. This supports the hypothesis that there is a correlation between the increased expression of defense-related genes and the enhanced resistance against pathogens in the ripening berry.

The composition, conjugation and quantity of anthocyanins in red varieties determine the color density and hue of the berry skin. Anthocyanins and PAs contribute to the astringency of wine and are also antioxidants with beneficial effects on human health [20]. Transcriptional regulation of the flavonoid pathway genes has been investigated mostly in *V. vinifera *varieties. Six MYB transcription factors (MYBA1, MYBA2, MYB5A, MYB5B, MYBPA1 and MYBPA2) are associated with the regulation of the structural genes in the flavonoid pathway. MYBA1 and MYBA2 play roles in the biosynthesis of anthocyanins by activating the promoter of *UFGT *[21-23], which catalyzes the last step of anthocyanin synthesis. MYB5A and MYB5B are involved in regulating several flavonoid biosynthesis steps [24]. MYBPA1and MYBPA2 regulate the last steps of pathways in the production of PAs [22,25].

Norton is considered a *V. aestivalis-*derived variety which produces high quality red wine that is comparable to wines made from *V. vinifera *grapes. Norton leaves accumulate high levels of salicylic acid (SA) and SA-associated defense genes in comparison with Cabernet Sauvignon. Abundant SA and high expression of SA-associated defense genes may equip Norton grape with a robust innate defense system against pathogens [26]. Furthermore, total amounts of anthocyanin and phenolic acid contents are significantly higher in Norton berries than in those of *V. vinifera *[27,28]. Similarly to other grape varieties that originate in North America, Norton berries develop exceptionally high levels of disease resistance, which enable viticulturists to grow this grape with minimal application of pesticides in regions with high disease pressure. Transcriptomics, proteomics, and metabolic profiles of berry development of *V. vinifera *varieties Cabernet Sauvignon and Pinot Noir have been studied and documented using Affymetrix GeneChips [7,8,15,29]. Consequently, the synthesis of flavonoids in the berry skin, and the expression and regulation of the underlying genes are well understood in *V. vinifera*. Little is known, however, about the regulation of the biosynthesis of flavonoid compounds in the berry skin of Norton. In this study, we analyzed the transcriptional profiles of over twenty thousand genes in Norton berry skin across six developmental stages using the second generation of Affymetrix *Vitis *microarrays (GRAPEGEN GenChip) [30]. We discovered a high coordination between the transcriptional regulation of key transcription factors and structural genes in the flavonoid biosynthesis pathway and the accumulation profiles of flavonoid compounds. Comparative analysis of key genes in flavonoid biosynthesis and of the main flavonoid compounds between Norton and Cabernet Sauvignon revealed variety-specific patterns of gene regulation and compound biosynthesis. The results from this study yield new knowledge on the distinct chemistry and characteristics of Norton grapes.

## Results and Discussion

### Discovery of differentially expressed genes during Norton berry skin development

Similarly to the berry development of *V. vinifera *varieties, the development of Norton berries is characterized by a two-stage growth pattern. Sugar accumulation began at the early stages and accelerated during véraison. Also following the pattern of *V. vinifera *berry development, the levels of titratable acidity dropped at stage 34 (at 66 days after bloom [DAB]) and continued to decrease until the berry was ripe. The descriptors of berry development, including berry diameter, titratable acidity and soluble solids, are presented in an accompanying paper (Ali *et al.*, in preparation). We started sampling on June 26, 2008 when the skin could be separated from the pulp of the berry. At this point, the berry was at stage 31 (17 DAB) on the Eichorn-Lorenz phenological scale. Subsequently, skin samples were taken at stages 33, 34, 35, 36, 37 and 38, corresponding to 37, 66, 71 (véraison), 85, 99, and 127 DAB. Skin tissue was frozen in liquid nitrogen and total RNA was extracted subsequently. The RNA was then labeled and hybridized to GRAPEGEN Affymetrix GeneChips. Processing of raw intensity values in CEL files and subsequent normalization and Median polishing were described in the paper (Ali *et al*., in preparation).

A Principal Component Analysis (PCA) of the eighteen arrays was performed to assess the similarity of expression values among the replicates (Additional File 1). The results from the PCA indicated a high degree of similarity among three biological replicates that were clustered tightly within the scatterplot. In addition, PCA showed that data of two proximal developmental stages were more similar to each other than data of distal developmental stages. There is a clear alignment and separation of developmental stages along the PC1 in the plot (Additional File 1). The eighteen sets of the data were then converted to z-scores and subjected to two-way unsupervised agglomerative cluster analysis (Additional File 2). This analysis showed that each stage represents a major branch which contains only the three biological replicate data for that stage. The results from these two analyses demonstrated that there is a good reproducibility among the three biological replicates and thus all data were included in the analysis. Pearson correlation coefficients between biological replicates were also calculated and were in the range of 0.9812 to 0.9976 (Additional File 3), further corroborating significant correlations between biological replicates in each developmental stage.

After the data of all eighteen arrays were processed and assessed for quality, the error-weighted intensity experiment definitions (EDs) were calculated by averaging the intensity of three biological replicates for each stage and then error-corrected using the Rosetta error model [31]. ANOVA was conducted on the error-weighted intensity of three biological replicates at each stage across six developmental stages with the Benjamini-Hochberg False Discovery Rate multiple test correction [32]. This resulted in the discovery of 15,823 probe sets that exhibited significant variations at the transcript levels between at least two developmental stages at *P *≤ 0.001 (Additional File 4). The differentially expressed probe sets comprise more than 78% of all probe sets on the microarray, indicating that a large number of genes represented on the array changed significantly at transcript levels at some points during berry development. To discover the genes whose transcript levels varied significantly from a baseline calculated from all six developmental stages, the intensity EDs of each probe set were divided by an error-weighted average of all six developmental stages. Under the criteria of absolute fold-change ≥2.0 in at least one developmental stage and having a LogRatio *P *-value ≤ 0.001 in at least one stage, we identified 3,352 probe sets (Additional File 5). We selected this group of the most significantly expressed genes for the subsequent analysis. The large number of transcripts that changed at expression levels corroborated earlier findings that genes of different functions were detected in the berry skin at the beginning of véraison and the later stages of ripening, reflecting the dramatic biochemical changes that take place during berry ripening [7,15].

### Cluster analysis of differentially expressed genes in Norton berry skin

We used the nucleotide sequence from which each set of probes was designed to acquire the best-matched GSVIVT ID in Genoscope (http://www.genoscope.cns.fr/externe/GenomeBrowser/Vitis/) or TC number in DFCI Grape Gene Index (http://compbio.dfci.harvard.edu/tgi/cgi-bin/tgi/gimain.pl?gudb=grape). The total of 3,352 probe sets represented 2,760 unique genes. We removed those probe sets where more than one probe set was assigned to the same GSVIVT ID or TC numbers but showed different expression patterns, and compiled them into a separate file for future analysis. At this time, it is not possible to discern what factors, such as alternatively spliced transcripts or degradation biases of the 5'-end and 3'-end portion of mRNA, influence the expression levels of these genes. We subjected the Log_2_-transformed fold-change of the remaining 2,359 unigenes to clustering by the *k*-means method. A total of 20 clusters were defined from this group of genes based on the figure of merit value (Additional File 6).

**Figure 1:**
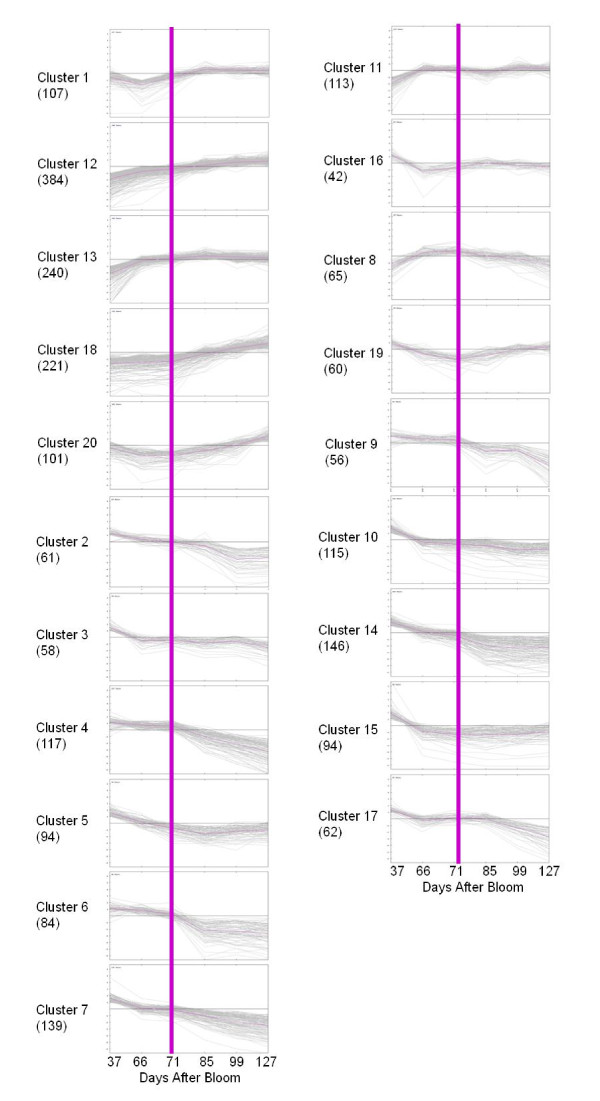
**Clustering of the expression profiles of 2,359 genes that were defined as significantly changed across the six developmental stages of Norton berry skin**. Clustering was performed using *k*-means statistics and 20 clusters were chosen for further analysis of transcriptional patterns. The number of genes in each cluster is listed in parenthesis. The X-axis indicates grape berry developmental stages in days after bloom (DAB); The Y-axis indicates the Log_2_-transformed fold-change of stage-specific intensity relative to the baseline intensity of each gene. The véraison phase is denoted by purple bar. A list of genes, their ChipID, Genoscope ID, putative function, Enzyme ID and pathway in Vitisnet for each cluster is included in Additional File 6.

Transcript abundance of these genes in cluster 1, 12, 13, 18 and 20 increased after véraison (Figure 1). These five clusters contained a total of 1,053 genes. Cluster 11 (113 genes) and Cluster 16 (42 genes) represented a pattern of transient increase and decrease, respectively, of transcript levels at the onset of véraison and subsequently unchanged post-véraison. The expression pattern of cluster 8 (65 genes) and cluster 19 (60 genes) was reciprocal. In cluster 8, transcript levels increased pre-véraison and decreased post-véraison. In cluster 19, transcript levels decreased at véraison, but increased both pre-véraison and post-véraison. The remaining eleven clusters included 1,026 genes and exhibited a pattern of steady decline post-véraison. The genes in each cluster are listed in Additional File 6.

### Developmental regulation of defense-related genes

A total of 48 differentially expressed genes were associated with defense, disease resistance, and hypersensitive response (Table 1). Among them, twenty one genes were up-regulated, and twenty five genes were down-regulated post-véraison. These defense-related genes include the well characterized polygalacturonase inhibiting protein (PGIP), dirigent protein, NBS-LRR, Non-race-specific disease resistance 1 (NDR1), powdery mildew resistant 5 (PMR5), and harpin-induced protein 1 genes.

**Table 1:** Transcriptional profiles of genes in Norton berry skin that are associated with defense pathways

**Cluster**^**A**^	Affymetrix ChipID	Genoscope ID	**Function (VitisNet)**^**B**^	KEGG	Pathway (VitisNet)
**Up-regulation post v**é**raison**					
1	VVTU11871_s_at	GSVIVT00025506001	Polygalacturonase inhibiting protein PGIP1	PGIP	Defense
12	VVTU6661_at	GSVIVT00005104001	Dirigent		Defense
18	VVTU13759_at	GSVIVT00038581001	Pathogenesis-related protein 1	PRP1	Defense
18	VVTU1755_at	GSVIVT00033081001	Pathogenesis protein 10.1		Defense
18	VVTU39372_at	GSVIVT00024739001	Dirigent protein		Defense
18	VVTU21514_x_at	GSVIVT00024741001	Dirigent protein		Defense
18	VVTU8656_at	GSVIVT00036870001	Epoxide hydrolase 2	3.3.2.10	Defense
13	VVTU10916_at	GSVIVT00018587001	Ripening induced protein		Defense response
20	VVTU4789_at	GSVIVT00007703001	NtPRp27 secretory protein		Defense response
1	VVTU10868_at	GSVIVT00037825001	Disease resistance protein		Disease resistance
18	VVTU16881_at	GSVIVT00028656001	Disease resistance protein (NBS-LRR class)		Disease resistance
20	VVTU7497_s_at	GSVIVT00000261001	Disease resistance protein (TIR-NBS class)		Disease resistance
20	VVTU36452_at	GSVIVT00038332001	TIR-NBS-LRR disease resistance		Disease resistance
12	VVTU40849_s_at	GSVIVT00030517001	Major latex protein 22		Disease resistance
12	VVTU35326_at	GSVIVT00002134001	Seed maturation protein PM41		Disease resistance
13	VVTU2601_at	GSVIVT00018817001	PMR5 (POWDERY MILDEW RESISTANT 5)		Disease resistance
20	VVTU9483_at	GSVIVT00000260001	TIR-NBS-LRR-TIR disease resistance protein		Disease resistance
20	VVTU2928_at	GSVIVT00021517001	Hairpin inducing protein 1-like 9		Hypersensitive response
20	VVTU37592_at	GSVIVT00023399001	Hairpin induced protein		Hypersensitive response
18	VVTU11329_at	GSVIVT00030027001	SP1L1 (SPIRAL1-LIKE1)		Pathogen
18	VVTU1632_at	GSVIVT00030524001	Bet v I allergen		Pathogenesis
**Up-down-up regulation**					
19	VVTU4500_s_at	GSVIVT00036464001	Viral-response family protein-like		Defense
19	VVTU7944_at	GSVIVT00016484001	BREVIS RADIX 4		Disease resistance
**Down-regulation post v**é**raison**					
9	VVTU3745_s_at	GSVIVT00024648001	Polygalacturonase inhibitor protein	PGIP	Defense
7	VVTU3256_at	GSVIVT00024747001	Dirigent protein pDIR9		Defense
14	VVTU4542_at	GSVIVT00016676001	Lachrymatory factor synthase		Defense
15	VVTU28352_at	GSVIVT00024745001	Dirigent protein		Defense
14	VVTU2350_at	GSVIVT00033031001	Epoxide hydrolase	3.3.2.10	Defense
17	VVTU2606_at	GSVIVT00025834001	Epoxide hydrolase 2	3.3.2.10	Defense
3	VVTU34452_at	GSVIVT00004842001	Disease resistance protein (TIR-NBS-LRR class)		Disease resistance
5	VVTU2751_s_at	GSVIVT00033825001	Disease resistance protein		Disease resistance
7	VVTU20455_at	GSVIVT00018767001	Receptor kinase TRKa		Disease resistance
7	VVTU21216_at	GSVIVT00020681001	Disease resistance protein (NBS-LRR class)		Disease resistance
14	VVTU10907_at	GSVIVT00011855001	HcrVf1 protein		Disease resistance
14	VVTU1732_at	GSVIVT00025424001	Disease resistance responsive		Disease resistance
14	VVTU34204_s_at	GSVIVT00025429001	Disease resistance responsive		Disease resistance
15	VVTU24464_at	GSVIVT00026768001	Disease resistance protein (CC-NBS-LRR class)		Disease resistance
2	VVTU52_at	GSVIVT00027396001	NDR1 (NON RACE-SPECIFIC DISEASE RESISTANCE)		Disease resistance
3	VVTU8917_at	GSVIVT00033069001	Major allergen Pru ar 1		Disease resistance
5	VVTU29478_at	GSVIVT00025399001	PMR5 (POWDERY MILDEW RESISTANT 5)		Disease resistance
9	VVTU5508_s_at	GSVIVT00033067001	Major cherry allergen Pru av 1.0202		Disease resistance
14	VVTU30737_at	GSVIVT00018816001	PMR5 (POWDERY MILDEW RESISTANT 5)		Disease resistance
3	VVTU2005_at	GSVIVT00026172001	Hairpin induced 1		Hypersensitive response
5	VVTU10307_x_at	GSVIVT00006738001	Hairpin induced 1		Hypersensitive response
14	VVTU14941_at	GSVIVT00034176001	Hairpin induced 1		Hypersensitive response
15	VVTU16087_at	GSVIVT00032401001	G protein protein gamma subunit (AGG2)		Pathogen defense
17	VVTU27983_at	GSVIVT00023169001	Mlo3	K08472	Pathogen defense
17	VVTU7548_x_at	GSVIVT00030529001	Bet v I allergen		Pathogenesis

^A ^Expression profiling of each cluster is shown in Figure 1. ^B ^Function annotation and pathway assignment of each gene were based on VitisNet (http://vitis-dormancy.sdstate.org/pathways.cfm)

Especially noticeable is the expression profile of the *PR-1 *gene, which is an indicator for the induction of local defense and systemic acquired resistance (SAR) in plants [33,34]. In grapevine, the *PR-1 *gene (GSVIVT00038581001) was induced by salicylic acid [35], and up-regulated after infection with the powdery mildew (PM) fungal pathogen *Erysiphe necator *[26]. Transcript levels of *PR-1 *increased progressively post-véraison in both Norton (cluster 18, Figure 1 and Table 1), and Cabernet Sauvignon [7,29]. The gene *AtWRKY75 *plays an important role in the activation of basal and resistance (*R*) gene-mediated resistance in Arabidopsis [36], and transcript levels of its grapevine ortholog increased in response to PM infection [26]. Interestingly, the grapevine *WRKY75 *ortholog was discovered in cluster 18. Four NBS-LRR genes were also identified in cluster 18, indicating these proteins are regulated developmentally in grape (Table 1). Plant NBS-LRR proteins are receptors that directly or indirectly recognize pathogen-deployed proteins, and this specific recognition triggers plant defense responses [37,38]. In some cases, they also play a role in the regulation of developmental pathways [39].

Five probe sets were annotated as thaumatin-like proteins and two as osmotins. Their transcript levels increased significantly in the late stages of Norton berry development (Additional File 5 and 6), as was shown previously in varieties of *V. vinifera *[7,29]. Thaumatin-like proteins inhibit spore germination and hyphal growth of *E. necator*, *Phomopsis viticola*, and *Botrytis cinerea *[40]. We found that transcript levels of five chitinase genes increased post-véraison in Norton berry skin (cluster 12, 13, 19, and 20). Transcript levels of basic class I (VCHIT1b) and a class III (VCH3) chitinase of grapevines increase in response to the chemical activators of SAR and are considered as markers of SAR [41]. Furthermore, enzymatic activities of chitinase and ß-1,3-glucanase also increase during berry development in the absence of pathogens [15]. Non-specific lipid transfer proteins (nsLTPs) belong to a family of small cystein-rich proteins that are induced in response to fungal elicitors and are associated with grapevine defense [42-44]. A possible LTP-jasmonic acid complex may protect grape berries against *B. cinerea *[42]. Transcripts of one probe set (GSVIVT00037486001) encoding VvLPT1, which are more prevalent in berry skin than in seeds [9], also increased steadily in Norton berries post-véraison (Cluster 1, Figure 1 and Additional File 6). In summary, differential expression of these defense-related genes indicates a developmentally regulated modulation of defense responses during ripening in Norton berry skin.

### Transcripts of stilbene synthase genes increased in Norton berry skin post-véraison

**Figure 2:**
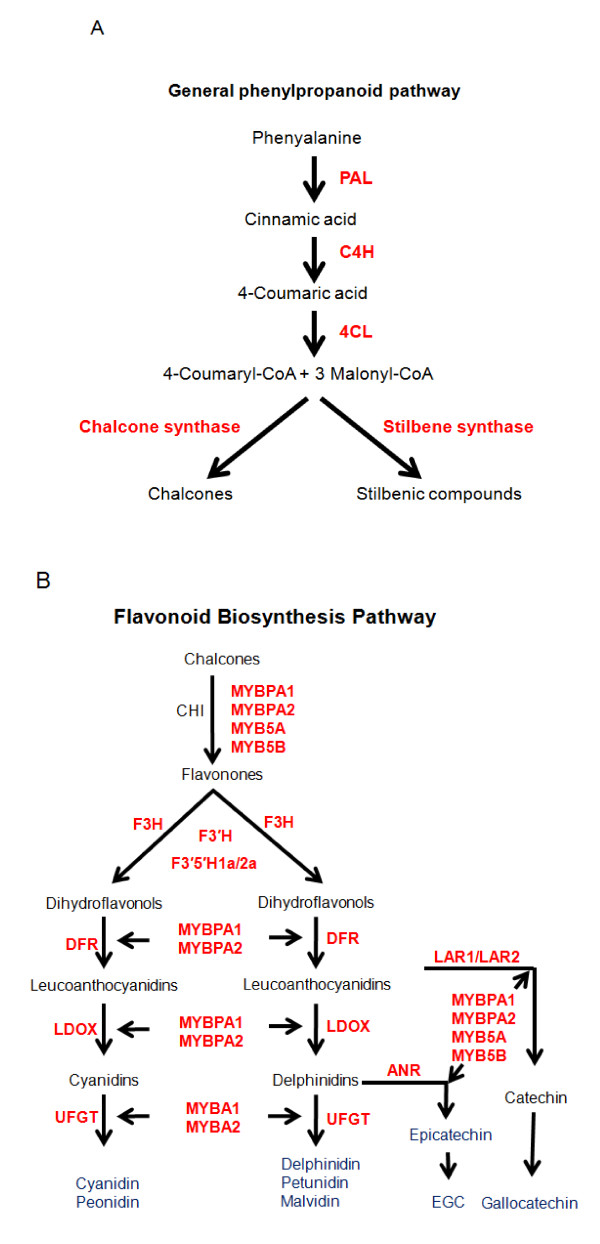
**Overview of the general phenylpropanoid pathway**. A: A simplified representation of the phenylpropanoid pathway leading to the production of chalcones and stilbenic compounds; B: The flavonoid biosynthesis pathway that leads to the production of anthocyanins and proanthocyanidins; six MYB transcription factors are indicated along the branches that are likely involved in the transcriptional regulation of the structural genes. PAL, phenylalanine ammonia-lyase; C4H, cinnamate 4-hydroxylase; 4CL, 4-coumarate-CoA ligase; CHI, chalcone isomerase; F3H, flavanone 3-hydroxylase; F3'H, flavonoid-3'-O-hydroxylase; F3'5'H, flavonoid-3',5'-hydroxylase; DFR, dihydroflavonol-4-reductase; LDOX, leucoanthocyanidin dioxygenase; UFGT, UDP-glucose:flavonoid-3-O-glucosyltransferase; ANR, anthocyanidin reductase; LAR, leucoanthocyanidin reductase; EGC, epigallocatechin.

The *cis*- and *trans*-piceid compounds of the stilbene family constitute a major group of phytoalexins in grapevines that are involved in the defense responses to pathogens [45]. They have been shown to have antifungal activities against several fungal pathogens including *Plasmopara viticola *[46] and *B. cinerea *[47,48]. They also exhibit antibacterial activity against *Xylella fastidiosa *[49], the pathogen of Pierce's disease on grapevine. In addition, stilbenic compounds possess anticancer and anti-inflammatory activities that have potential benefits to human health [50]. Stilbene synthase (STS) is the key enzyme that catalyzes the formation of 3', 4', 5'-trihydroxystilbene (resveratrol) via the condensation of one 4-coumaroyl-CoA and three malonyl-CoA molecules (Figure 2A). This condensation reaction represents a branch point in the phenylpropanoid pathway, at which CHS channels 4-coumaroyl-CoA molecules towards flavonoid synthesis and STS towards stilbene synthesis.

Grape berry skin is the main tissue where the synthesis of stilbenes occurs [51]. STS was found to be localized mostly in the cell wall of hypodermal cells in the exocarp, which is in agreement with the detection of stilbenic compounds mainly in the exocarp during berry development [51]. It was also demonstrated that stilbenic compounds and transcripts of the key genes *PAL*, *4CL*, and *STS *accumulated progressively in ripening berries of Pinot Noir [52] and Corvina [53]. The composition of stilbenic compounds differs significantly among grape varieties. Mature berries of Pinot Noir contain the highest levels of stilbenes, while the stilbene content of Cabernet Sauvignon berries is ranked 41st among 48 red-skinned grapes [52]. There is a high correlation between the transcript levels of *PAL*, *4CL*, and *STS *and the abundance of stilbenic compounds in grape varieties [52,53]. We found that six of the ten paralogous *STS *genes on the GrapeGen Chip are grouped into clusters 18 and 20, and the transcripts of these genes increased steadily and significantly post-véraison (Figure 1). Interestingly, *PAL *and *4CL *were also found in clusters 18 and 20, in which transcripts of these genes significantly increased in the final two stages (Figure 1). Highly coordinated expression of *PAL*, *4CL*, and *STS *post-véraison strongly supports the conclusion that the stilbene biosynthesis pathway is up-regulated during the development of Norton berry skin. In our previous microarray analysis of the pathogen-induced transcriptome in grapevines, we discovered that *STS *genes were strongly induced in response to PM infection [26]. These results confirm that stilbenes, together with other phytoalexins and defense-related proteins, are part of the defense weaponry for protecting berries from pathogen attacks. This defense strategy appears to be developmentally regulated in Norton berry skin.

### Coordinated expression of the phenylpropanoid and flavonoid pathways

Results of previous microarray analyses of tissue-specific transcriptomes demonstrated that the majority of genes encoding enzymes in the biosynthesis of flavonoids, lignin, anthocyanins and proanthocyanidins were expressed preferentially in the berry skin of grapevine [9]. These genes include *PAL*, *C4H*, and *4CL*, encoding key enzymes which catalyze the first three steps of the phenylpropanoid pathway (Figure 2A). The present microarray analysis also showed that transcripts of three *PAL *genes and one *4CL *gene increased significantly in Norton berry skin post-véraison (Table 2). The increasing levels of *PAL *and *4CL *transcripts most likely led to higher accumulation of the substrate 4-coumaryl-CoA for the down-stream pathways. This trend coordinates well with the transcriptional regulation of chalcone synthase (CHS) (GSVIVT00037967001), six STSs, DFR (GSVIVT00014584001) and GSVIVT00036313001), LDOX (GSVIVT00001063001), and UFGT (GSVIVT00014047001). Transcripts of these genes increased post-véraison (Table 2). This up-regulation of the phenylpropanoid pathway in the skin of the ripening berry has also been observed in Cabernet Sauvignon [15]. Interestingly, the genes that were expressed at the highest level in Cabernet Sauvignon encoded enzymes mostly in the flavonoid biosynthesis pathway downstream of PAL, C4H and 4CL.

**Table 2:** Transcriptional profiles of genes in Norton berry skin that are associated with secondary metabolism

**Cluster**^**A**^	Affymetrix ChipID	Genoscope ID	**Function (VitisNet)**^**B**^	KEGG	Pathway (VitisNet)
**1**	**VVTU703_s_at**	**GSVIVT00018175001**	**Phenylalanine ammonia lyase 2 (PAL2)**	**4.3.1.5**	**Phenylpropanoid**
**1**	**VVTU12705_s_at**	**GSVIVT00024561001**	**Phenylalanine ammonia lyase (PAL)**	**4.3.1.5**	**Phenylpropanoid**
**18**	**VVTU26285_at**	**GSVIVT00013936001**	**Phenylalanine ammonia lyase (PAL)**	**4.3.1.5**	**Phenylpropanoid**
*4*	*VVTU39693_at*	*GSVIVT00008924001*	*Cinnamyl alcohol dehydrogenase (CAD)*	*1.1.1.195*	*Phenylpropanoid*
*6*	*VVTU2766_at*	*GSVIVT00011484001*	*Sinapyl alcohol dehydrogenase (SAD)*	*1.1.1.195*	*Phenylpropanoid*
*10*	*VVTU14855_at*	*GSVIVT00024588001*	*Cinnamyl alcohol dehydrogenase (CAD)*	*1.1.1.195*	*Phenylpropanoid*
**20**	**VVTU21888_at**	**GSVIVT00011639001**	**Cinnamyl alcohol dehydrogenase (CAD)**	**1.1.1.195**	**Phenylpropanoid**
*2*	*VVTU13147_s_at*	*GSVIVT00013987001*	*Cinnamoyl-CoA reductase (CCR)*	*1.2.1.44*	*Phenylpropanoid*
*7*	*VVTU12930_s_at*	*GSVIVT00033763001*	*Cinnamoyl-CoA reductase (CCR)*	*1.2.1.44*	*Phenylpropanoid*
**12**	**VVTU3517_at**	**GSVIVT00015738001**	**Cinnamoyl-CoA reductase (CCR)**	**1.2.1.44**	**Phenylpropanoid**
**13**	**VVTU914_at**	**GSVIVT00038153001**	**Cinnamoyl-CoA reductase (CCR)**	**1.2.1.44**	**Phenylpropanoid**
**20**	**VVTU15680_at**	**GSVIVT00020726001**	**Cinnamoyl-CoA reductase (CCR)**	**1.2.1.44**	**Phenylpropanoid**
**13**	**VVTU4884_at**	**GSVIVT00002825001**	**Caffeoyl-CoA O-methyltransferase (CCoAOMT)**	**2.1.1.104**	**Phenylpropanoid**
**18**	**VVTU36108_at**	**GSVIVT00025990001**	**Caffeic acid O-methyltransferase (CAOMT)**	**2.1.1.68**	**Phenylpropanoid**
**18**	**VVTU6966_s_at**	**GSVIVT00026179001**	**Caffeate 3-O-methyltransferase 1 (COMT)**	**2.1.1.68**	**Phenylpropanoid**
**12**	**VVTU34546_at**	**GSVIVT00009234001**	**Stilbene synthase (STS)**	**2.3.1.95**	**Phenylpropanoid**
**18**	**VVTU34913_at**	**GSVIVT00007353001**	**Stilbene synthase (STS)**	**2.3.1.95**	**Phenylpropanoid**
**18**	**VVTU34551_x_at**	**GSVIVT00031875001**	**Stilbene synthase (STS)**	**2.3.1.95**	**Phenylpropanoid**
**18**	**VVTU11765_at**	**GSVIVT00004049001**	**Stilbene synthase (STS)**	**2.3.1.95**	**Phenylpropanoid**
**18**	**VVTU7619_x_at**	**GSVIVT00005196001**	**Stilbene synthase (STS)**	**2.3.1.95**	**Phenylpropanoid**
**18**	**VVTU2775_x_at**	**GSVIVT00007358001**	**Stilbene synthase (STS)**	**2.3.1.95**	**Phenylpropanoid**
**18**	**VVTU18886_x_at**	**GSVIVT00007364001**	**Stilbene synthase (STS)**	**2.3.1.95**	**Phenylpropanoid**
**18**	**VVTU6035_x_at**	**GSVIVT00009221001**	**Stilbene synthase (STS)**	**2.3.1.95**	**Phenylpropanoid**
**20**	**VVTU26310_s_at**	**GSVIVT00031885001**	**Stilbene synthase (STS)**	**2.3.1.95**	**Phenylpropanoid**
**20**	**VVTU2671_at**	**GSVIVT00009225001**	**Stilbene synthase (STS)**	**2.3.1.95**	**Phenylpropanoid**
*7*	*VVTU15752_at*	*GSVIVT00002505001*	*Pinoresinol forming dirigent protein*	*DIRPR*	*Phenylpropanoid*
16	VVTU8264_at	GSVIVT00023306001	p-Coumaroyl shikimate 3'-hydroxylase isoform 1	K09754	Phenylpropanoid
*14*	*VVTU25372_at*	*GSVIVT00017649001*	*Ferulate 5-hydroxylase (F5H)*	*K09755*	*Phenylpropanoid*
**18**	**VVTU8974_at**	**GSVIVT00036840001**	**Ferulate 5-hydroxylase (F5H)**	**K09755**	**Phenylpropanoid**
*14*	*VVTU34012_at*	*GSVIVT00017653001*	*Ferulate 5-hydroxylase (F5H)*	*K09755*	*Phenylpropanoid*
*2*	*VVTU6513_s_at*	*GSVIVT00038750001*	*Pinoresinol-lariciresinol reductase*	*PLR*	*Phenylpropanoid*
*15*	*VVTU15529_s_at*	*GSVIVT00021542001*	*Secoisolariciresinol dehydrogenase*	*SIRD*	*Phenylpropanoid*
**20**	**VVTU2645_at**	**GSVIVT00031383001**	**4-Coumarate-CoA ligase 2 (4CL)**	**6.2.1.12**	**Phenylpropanoid**
**1**	VVTU17924_s_at*	**GSVIVT00014584001**	**Dihydroflavonol 4-reductase (DFR)**	**1.1.1.219**	**Flavonoid**
**12**	**VVTU14294_at**	**GSVIVT00036313001**	**Dihydroflavonol-4-reductase (DFR)**	**1.1.1.219**	**Flavonoid**
**13**	VVTU36178_s_at*	**GSVIVT00001063001**	**Leucoanthocyanidin dioxgenase (LDOX)**	**1.14.11.19**	**Flavonoid**
*11*	*VVTU9714_at*	*GSVIVT00007249001*	*Flavonol synthase (FLS)*	*1.14.11.23*	*Flavonoid*
**13**	**VVTU33390_s_at**	**GSVIVT00031249001**	**Flavonol synthase (FLS)**	**1.14.11.23**	**Flavonoid**
*14*	*VVTU13981_at*	*GSVIVT00007247001*	*Flavonol synthase (FLS)*	*1.14.11.23*	*Flavonoid*
**18**	**VVTU2456_s_at**	**GSVIVT00015347001**	**Flavonol synthase (FLS)**	**1.14.11.23**	**Flavonoid**
*10*	*VVTU16387_at*	*GSVIVT00015842001*	*Naringenin,2-oxoglutarate 3-dioxygenase*	*1.14.11.9*	*Flavonoid*
**13**	**VVTU39787_s_at**	**GSVIVT00036784001**	**Flavanone 3-hydroxylase (F3H)**	**1.14.11.9**	**Flavonoid**
**13**	**VVTU37475_at**	**GSVIVT00037165001**	**Flavanone 3-hydroxylase (F3H)**	**1.14.11.9**	**Flavonoid**
**1**	**VVTU7778_at**	**GSVIVT00034070001**	**Flavonoid 3-monooxygenase**	**1.14.13.21**	**Flavonoid**
*4*	*VVTU6932_at*	*GSVIVT00016437001*	*Flavonoid 3-monooxygenase*	*1.14.13.21*	*Flavonoid*
*4*	*VVTU25410_s_at*	*GSVIVT00036466001*	*Flavonoid 3-monooxygenase*	*1.14.13.21*	*Flavonoid*
*7*	*VVTU6362_at*	*GSVIVT00017654001*	*Flavonoid 3-monooxygenase*	*1.14.13.21*	*Flavonoid*
**13**	**VVTU35884_at**	**GSVIVT00022300001**	**Flavonoid 3',5'-hydroxylase (F3'5'H)**	**1.14.13.88**	**Flavonoid**
*10*	VVTU13083_at*	*GSVIVT00005344001*	*Anthocyanidin reductase (ANR)*	*1.3.1.77*	*Flavonoid*
**13**	**VVTU9453_at**	**GSVIVT00000479001**	**Quercetin 3-O-methyltransferase 1**	**2.1.1.76**	**Flavonoid**
**1**	**VVTU39820_s_at**	**GSVIVT00037967001**	**Chalcone synthase(CHS)**	**2.3.1.74**	**Flavonoid**
*5*	*VVTU15193_at*	*GSVIVT00003466001*	*UDP-glucose:flavonoid 7-O-glucosyltransferase (UFGT)*	*2.4.1.237*	*Flavonoid*
*14*	*VVTU22370_at*	*GSVIVT00033493001*	*UDP-glucose:flavonoid 7-O-glucosyltransferase (UFGT)*	*2.4.1.237*	*Flavonoid*
13	VVTU17578_s_at*	GSVIVT00014047001	UDP-glucose:flavonoid 3-O-glucosyltransferase (UFGT)	2.4.1.91	Flavonoid
*3*	*VVTU15110_at*	*GSVIVT00001621001*	*Flavonol 3-sulfotransferase*	*2.8.2.25*	*Flavonoid*
**1**	**VVTU3684_s_at**	**GSVIVT00029440001**	**Chalcone flavanone isomerase (CHI)**	**5.5.1.6**	**Flavonoid**
*17*	*VVTU563_at*	*GSVIVT00020652001*	*Chalcone isomerase (CHI)*	*5.5.1.6*	*Flavonoid*
*10*	*VVTU9073_x_at*	*GSVIVT00009968001*	*UDP-glucose: anthocyanidin 5,3-O-glucosyltransferase*	*2.4.1.238*	*Flavonoid*
**12**	**VVTU24324_at**	**GSVIVT00024127001**	**Anthocyanidin 3-O-glucosyltransferase**	**2.4.1.115**	**Anthocyanin**
**18**	**VVTU35521_at**	**GSVIVT00024993001**	**Anthocyanidin 3-O-glucosyltransferase**	**2.4.1.115**	**Anthocyanin**
19	VVTU15768_at	GSVIVT00037558001	Anthocyanidin 3-O-glucosyltransferase	2.4.1.115	Anthocyanin
**20**	**VVTU14014_at**	**GSVIVT00005849001**	**Anthocyanidin 3-O-glucosyltransferase**	**2.4.1.115**	**Anthocyanin**
*7*	*VVTU8698_at*	*GSVIVT00008206001*	*Anthocyanidin rhamnosyl-transferase*	*RHATR*	*Anthocyanin*
8	VVTU10613_at	GSVIVT00026922001	Anthocyanidin rhamnosyl-transferase	RHATR	Anthocyanin
**13**	**VVTU7774_at**	**GSVIVT00011809001**	**UDP-rhamnose/rhamnosyltransferase**	**RHATR**	**Anthocyanin**
*5*	*VVTU8944_x_at*	*GSVIVT00001860001*	*UDP-glucose: anthocyanidin 5,3-O-glucosyltransferase*	*RHGT1*	*Anthocyanin*
**12**	**VVTU14620_at**	**GSVIVT00001853001**	**UDP-glucose: anthocyanidin 5,3-O-glucosyltransferase**	**RHGT1**	**Anthocyanin**
**16**	**VVTU15845_at**	**GSVIVT00001851001**	**UDP-glucose: anthocyanidin 5,3-O-glucosyltransferase**	**RHGT1**	**Anthocyanin**
*17*	*VVTU15902_at*	*GSVIVT00001859001*	*UDP-glucose: anthocyanidin 5,3-O-glucosyltransferase*	*RHGT1*	*Anthocyanin*
**18**	**VVTU36907_at**	**GSVIVT00024130001**	**UDP-glucose: anthocyanidin 5,3-O-glucosyltransferase**	**RHGT1**	**Anthocyanin**
*3*	*VVTU5076_s_at*	*GSVIVT00033502001*	*UDP-glucoronosyl/UDP-glucosyl transferase UGT75C1*	*UGT75C1*	*Anthocyanin*
*15*	*VVTU38572_at*	*GSVIVT00025511001*	*CYP93A1 2-hydroxyisoflavanone synthase*	*1.14.13.86*	*Isoflavonoid*
**13**	**VVTU2075_at**	**GSVIVT00019588001**	**CYP81E1 Isoflavone 2'-hydroxylase**	**1.14.13.89**	**Isoflavonoid**
**20**	**VVTU22627_at**	**GSVIVT00019595001**	**CYP81E1 Isoflavone 2'-hydroxylase**	**1.14.13.89**	**Isoflavonoid**
*4*	*VVTU3973_at*	*GSVIVT00026339001*	*2'-hydroxy isoflavone/dihydroflavonol reductase*	*1.3.1.45*	*Isoflavonoid*
8	VVTU6973_at	GSVIVT00003030001	Isoflavone methyltransferase	2.1.1.46	Isoflavonoid

^A ^Clusters in bold exhibit steady increase of transcript abundance post véraison; Clusters in italics show decrease of transcript abundance post véraison. Expression profiling of each cluster is shown in Figure 1. ^B ^Function annotation and pathway assignment of each gene were based on VitisNet (http://vitis-dormancy.sdstate.org/pathways.cfm). The genes (DFR, LDOX, ANR, UFGT) with asterisk have the same GSVIVT ID and display similar expression profiling as in qPCR.

After we had compared the previous microarray analysis of Cabernet Sauvignon berry development [7] with the present results in Norton (Table 2), we discovered that the two grape varieties share eight genes that are differentially expressed in the flavonoid pathway. Particularly interesting is the finding that transcripts of *F3H *(GSVIVT00036784001), flavonol synthase (*FLS*) (GSVIVT00015347001), and *CHS *(GSVIVT00037967001) decreased progressively during Cabernet Sauvignon berry development, but increased steadily in Norton.

### Transcription profiles of flavonoid biosynthesis genes differ in the two varieties

**Figure 3:**
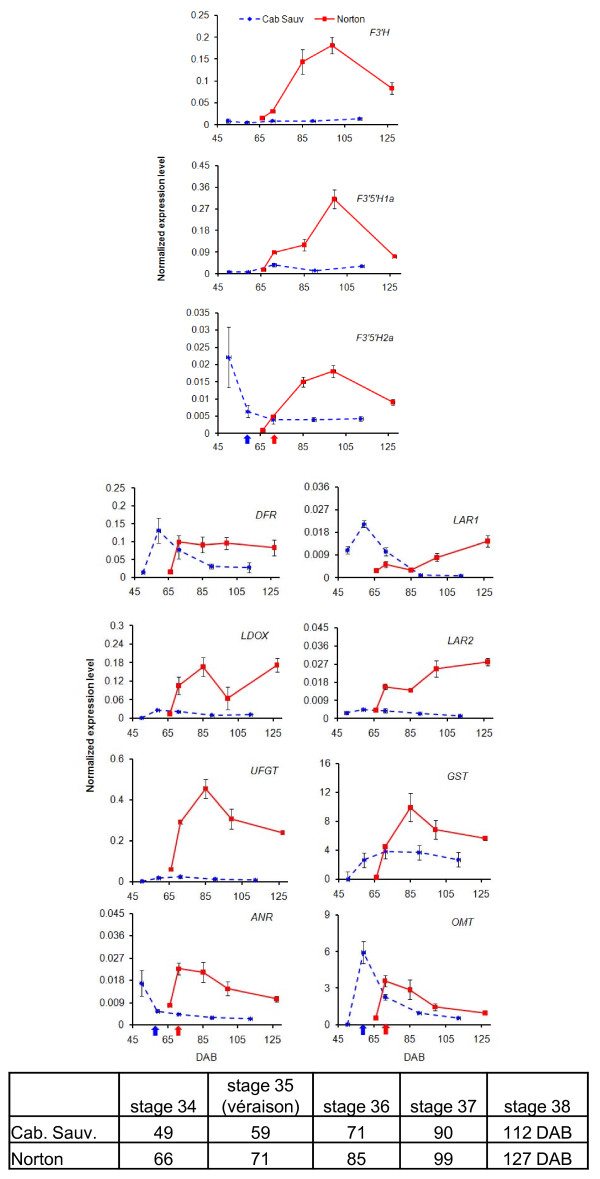
**Quantitative real-time (qPCR) assay of transcript abundance of the structural genes *F3'H*, *F3'5'H1a, F3'5'H2a, DFR*, *LDOX*, *UFGT*, *ANR*, *LAR1*, *LAR2*, *GST *and *OMT *in the flavonoid biosynthesis pathway during *Vitis vinifera *'Cabernet Sauvignon' (blue dashed line) and *V. aestivalis *'Norton' (red solid line) berry skin development**. Cabernet Sauvignon berry skin were collected at 49, 59 (véraison, blue arrow), 71, 90 and 112 days after bloom (DAB), and Norton berry skin at 66, 71 (véraison, red arrow), 85, 99 and 127 DAB. Transcript abundance of each gene was normalized by the level of an actin gene. Bars indicate standard error of three biological replicates at each sampling time-point.

The differential expression of flavonoid biosynthesis genes in Norton berry skin development prompted us to compare the transcript abundance of the most relevant genes in Norton with those in Cabernet Sauvignon. We conducted qPCR assays to compare transcript levels of eleven genes between the two varieties (Additional File 7). We chose these eleven genes based on their key roles in the pathway that F3'H, F3'5'H-1a and -2a, DFR, LDOX, and UFGT are involved in biosynthesis of anthocyanins while ANR and LAR1/2 catalyze PA synthesis (Figure 2B). Expression of the eleven genes exhibited distinctive patterns between the two varieties (Figure 3).

Transcripts of *F3'H, F3'5'H1a *and *F3'5'H2a *reached maximum levels at 99 DAB in Norton, and were significantly higher in Norton than in Cabernet Sauvignon post-véraison. Transcripts of *DFR *increased to the highest levels at véraison in both varieties, and then declined sharply in Cabernet Sauvignon, but remained at the same levels throughout the ripening stages in Norton. Transcripts of *LDOX *were very low in Cabernet Sauvignon, but in Norton they increased to a peak at 85 DAB, declined at 99 DAB, and then bounced back to the same levels at 127 DAB as at 85 DAB. *UFGT *transcript levels reached a maximum at 99 DAB, and also were significantly higher in Norton than in Cabernet Sauvignon (Figure 3).

Transcripts of *ANR *attained peak levels at véraison, and declined gradually in Norton, but were significantly higher in Norton than in Cabernet Sauvignon post-véraison. Transcripts of *LAR1 *were the most abundant at véraison, significantly higher in Cabernet Sauvignon than in Norton, and then declined to be barely detectable in the final two stages in Cabernet Sauvignon. In Norton, *LAR1 *transcript levels increased steadily after 85 DAB. On the other hand, *LAR2 *transcripts increased, and were also more abundant in Norton than in Cabernet Sauvignon post-véraison (Figure 3).

Taken together, transcripts of all eleven genes accumulated more abundantly in Norton after véraison, suggesting that the biosynthesis of flavonoid compounds remains highly activated in the skin of Norton berries post-véraison.

### Expression pattern of GST and OMT

In plants, *GSTs *consist of a large, complex gene family and play important roles in anthocyanin transport to or storage in the vacuole [54]. They conjugate the tripeptide glutathione to a variety of electrophilic compounds, thus limiting damaging effects of reactive oxygen species [55,56]. RNA-seq analysis showed that transcripts of 64 of the predicted 87 *GST*s in grapevine were detected during berry development of the grape variety 'Corvina' [57]. However, the specific roles of the individual *GSTs *were not clear. Four *GST *isoforms were identified in cell suspension cultures of grapevine. Two of them were highly expressed and involved in anthocyanin accumulation or transport into the vacuole [58]. One grapevine *GST *(GSVIVT00023496001) gene was well-characterized [54], and was chosen for qPCR analysis of this gene family during berry skin development. We found that transcript levels of this *GST *gene reached a peak at 85 DAB and declined slightly post-véraison, and were more abundant in Norton than in Cabernet Sauvignon berry skin (Figure 3). It is speculated that the difference in transcript levels of *GST *genes between the two varieties may lead to accumulation of more anthocyanins in the vacuoles of Norton berry skin cells than in those of Cabernet Sauvignon.

The methylation of phenolic compounds, as catalyzed by O-methyltransferases (*OMTs*), is an important step in flavonoid metabolism [59]. For example, caffeoyl CoA and caffeic acid *OMTs *are able to methylate lignin precursors [60,61]. On the basis of substrate specificity and function in stabilizing phenolic products, plant OMTs have been classified into various categories. Increasing evidence suggests that the expression of *OMT *genes is correlated with the accumulation of methylated anthocyanins in grapevines [62-64]. The qPCR results show that one *OMT *(GSVIVT00002831001) of grapevine was highly induced post-véraison when anthocyanins accumulated in both Cabernet Sauvignon and Norton. Transcript levels of this grapevine *OMT *were the highest at véraison, significantly higher in Cabernet Sauvignon than in Norton, and then declined gradually towards harvest (Figure 3). It is yet to be determined if this difference at transcript levels of this particular *OMT *could result in the production of different types of anthocyanin derivatives.

### Expression patterns of *MYB *transcription factors are unique in each variety

**Figure 4:**
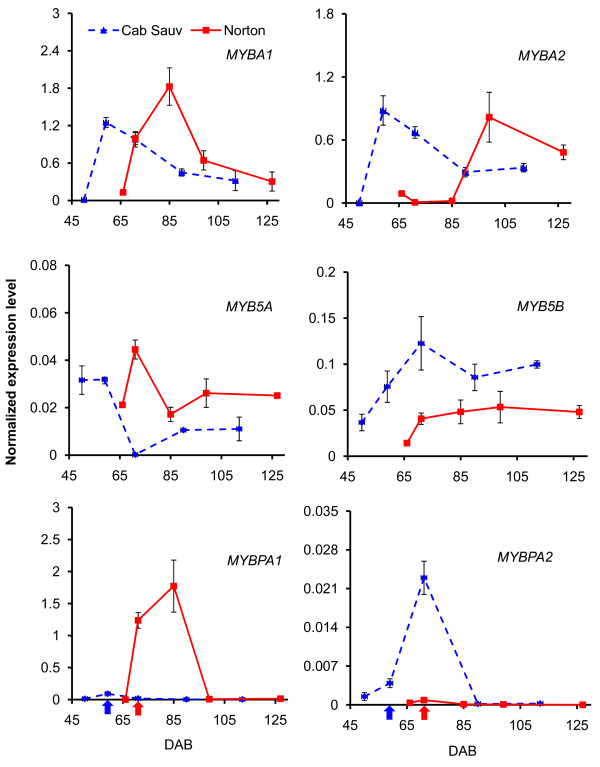
**Quantitative real-time (qPCR) assay of transcript levels of the six transcription factor genes *MYBA1*, *MYBA2*, *MYB5A*, *MYB5B*, *MYBPA1 *and *MYBPA2 *that regulate the flavonoid pathway in berry skin across five developmental stages of *V. vinifera *'Cabernet Sauvignon' (blue dashed line) and *V. aestivalis *'Norton' (red solid line)**. Cabernet Sauvignon berry skin were collected at 49, 59 (véraison, blue arrow), 71, 90 and 112 days after bloom (DAB), and Norton berry skin at 66, 71 (véraison, red arrow), 85, 99 and 127 DAB. Transcript abundance of each gene was normalized by the level of an actin gene. Bars indicate standard error of three biological replicates at each sampling time-point.

To investigate transcriptional regulation of the flavonoid pathway during berry skin development, we analyzed the transcript levels of six genes encoding MYB transcription factors (*MYBA1*, *MYBA2*, *MYBPA1*, *MYBPA2*, *MYB5A *and *MYB5B*) by qPCR (Additional File 7). All transcription factor genes assayed were expressed at some stages of berry skin development, but the expression patterns of some of them were distinct between the two varieties (Figure 4).

Expression profiles of *MYBA1 *and *MYBA2 *are very similar between the two varieties. *MYBA1 *transcripts reached peak levels at 85 DAB after véraison in Norton and then declined and remained low. Similarly, the transcripts of *MYBA1 *reached the highest level at 59 DAB (véraison) and decreased gradually post-véraison in Cabernet Sauvignon. *MYBA2 *transcripts also reached the highest level at 59 DAB, and then decreased until 112 DAB in Cabernet Sauvignon. In contrast, in Norton *MYBA2 *transcripts reached the highest level at 99 DAB.

The transcript profiles of *MYB5A *and *MYB5B *were similar during all of berry skin development, with high levels at véraison in both varieties. *MYB5A *transcript levels are slightly higher in Norton than in Cabernet Sauvignon while transcript levels of *MYB5B *are higher at all developmental stages in Cabernet Sauvignon than in Norton. The transcripts of *MYBPA1 *in Norton increased sharply from 66 to 71 DAB (véraison), reached the highest level at 85 DAB, and then declined to a barely detectable level. The transcript levels of *MYBPA1 *in Cabernet Sauvignon, on the other hand, remained low throughout berry development. In contrast, *MYBPA2 *transcripts reached maximum levels at 71 DAB in Cabernet Sauvignon, while they remained steadily low in Norton throughout berry development. The results suggest that MYBPA1 may play a more prominent role in Norton than in Cabernet Sauvignon whereas MYBPA2 in Cabernet Sauvignon than in Norton in the regulation of PA biosynthesis. The variety-specific regulation of MYBPAs warrants further functional analysis of their regulatory elements.

### Proanthocyanidin and anthocyanin profiles in berry skin of Norton and Cabernet Sauvignon

**Figure 5:**
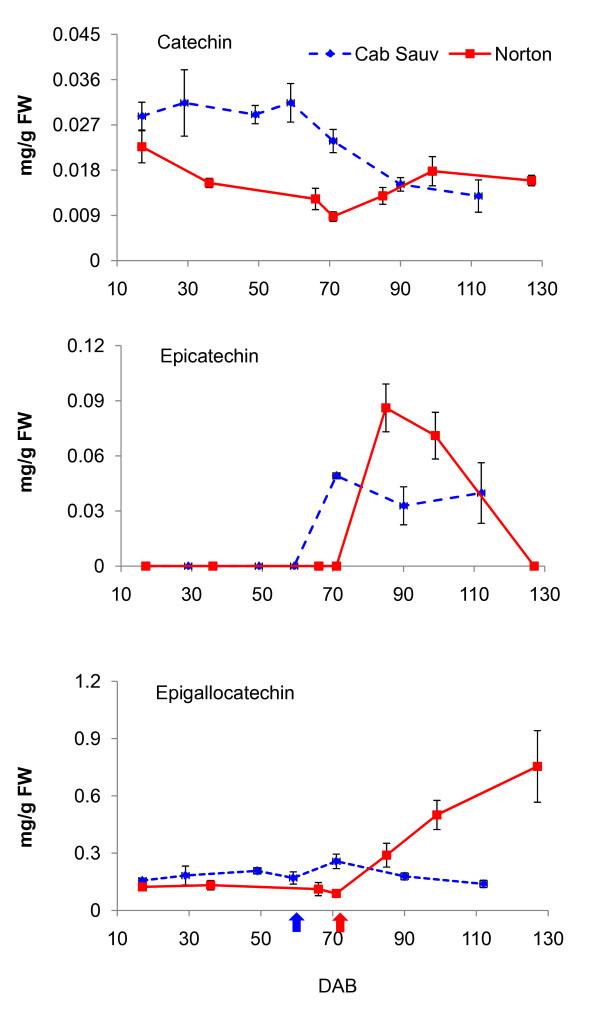
**Accumulation kinetics of the proanthocyanidins catechin, epicatechin, and epigallocatechin during *V. vinifera *'Cabernet Sauvignon' (blue dashed line) and *V. aestivalis *'Norton' (red solid line) berry skin development**. Cabernet Sauvignon berry skin were collected at 49, 59 (véraison, blue arrow), 71, 90 and 112 days after bloom (DAB), and Norton berry skin at 66, 71 (véraison, red arrow), 85, 99 and 127 DAB. Bars indicate standard error of three biological replicates per sample.

To match gene expression patterns with flavonoid profiles, we analyzed the accumulation of the flavan-3-ols catechin, epicatechin, epigallocatechin (EGC), and epicatechin gallate (ECG) in berry skin across seven developmental stages (Figure 5). Norton and Cabernet Sauvignon have comparative levels of catechin at 17 DAB. In Cabernet Sauvignon, catechin levels remained high until just after véraison, whereas in Norton, catechin dropped to the lowest levels at 71 DAB (véraison) and then rose until 127 DAB. Epicatechin was not detected in either variety until véraison, but was detectable in Norton at 85 and 99 DAB as well as in Cabernet Sauvignon post-véraison. EGC levels remained steady in Cabernet Sauvignon throughout berry development, but increased steadily in Norton until 127 DAB. ECG was detected only in Cabernet Sauvignon (data not shown).

**Figure 6:**
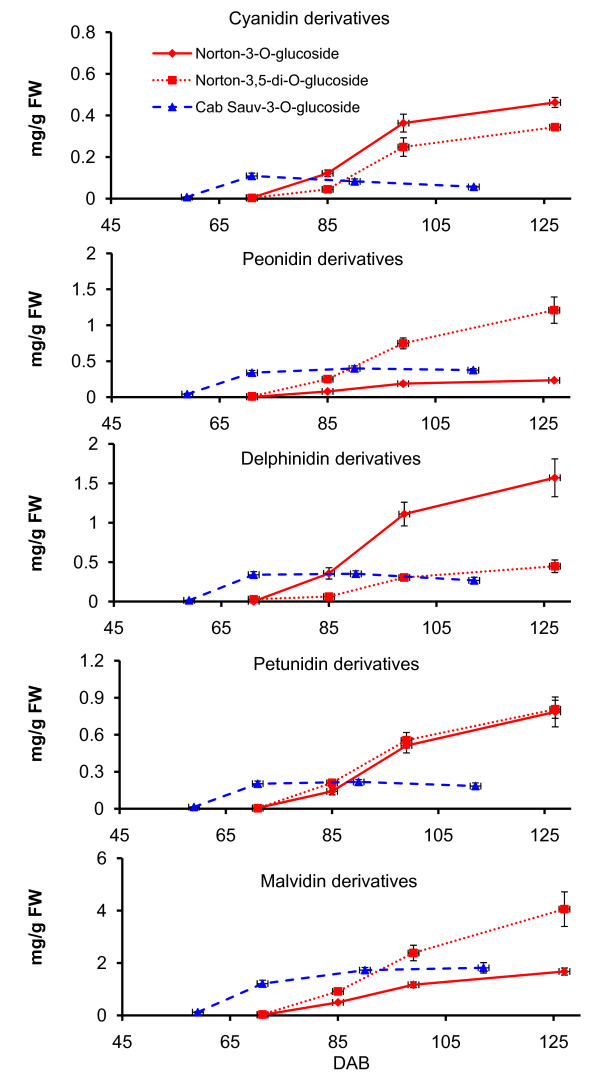
**Accumulation kinetics of the anthocyanidin derivatives cyanidin, peonidin, delphinidin, petunidin and malvidin glucosides during *V. vinifera *'Cabernet Sauvignon' (blue dashed line) and *V. aestivalis *'Norton' (red solid line) berry skin development**. Cabernet Sauvignon berry skin were collected at 49, 59 (véraison), 71, 90 and 112 days after bloom (DAB), and Norton berry skin at 66, 71 (véraison), 85, 99 and 127 DAB. Bars indicate standard error of three biological replicates per sample.

We analyzed the accumulation profiles of five anthocyanin derivatives (cyanidin-, peonidin-, delphinidin-, petunidin- and malvidin-monoglucoside/diglucoside) at four post-véraison stages of berry skin for both varieties by high performance liquid chromatography (HPLC) (Figure 6). Accumulation patterns of the five anthocyanins in Cabernet Sauvignon berry skin in the present study are remarkably similar to the previous observations in Cabernet Sauvignon under different climate and environmental conditions [65]. The accumulation of the five anthocyanins begins at véraison, and leads to much higher levels in Norton than in Cabernet Sauvignon at harvest (Figure 6).

In agreement with previous results that diglucoside derivatives of anthocyanins are found in *Vitis *species of North American origin [66], anthocyanin-diglucosides are highly abundant and contribute a major portion to the total anthocyanin content in Norton berry skin (Figure 6). Interestingly, the amounts of monoglucoside derivatives of malvidin and peonidin are not significantly different between Norton at 127 DAB, and Cabernet Sauvignon at 112 DAB. Diglucoside derivatives of peonidin and malvidin accumulated to significantly higher levels than their respective monoglucoside derivatives in Norton (Figure 6). Malvidin-diglucoside is the major anthocyanin in Norton while malvidin-monoglucoside contributes primarily to anthocyanin in Cabernet Sauvignon. The five anthocyanin derivatives reached their highest levels in Cabernet Sauvignon after véraison and remained steady until 112 DAB; whereas in Norton they continued to increase steadily until harvest at 127 DAB.

### Norton accumulates a broader spectrum of anthocyanins than Cabernet Sauvignon

**Figure 7:**
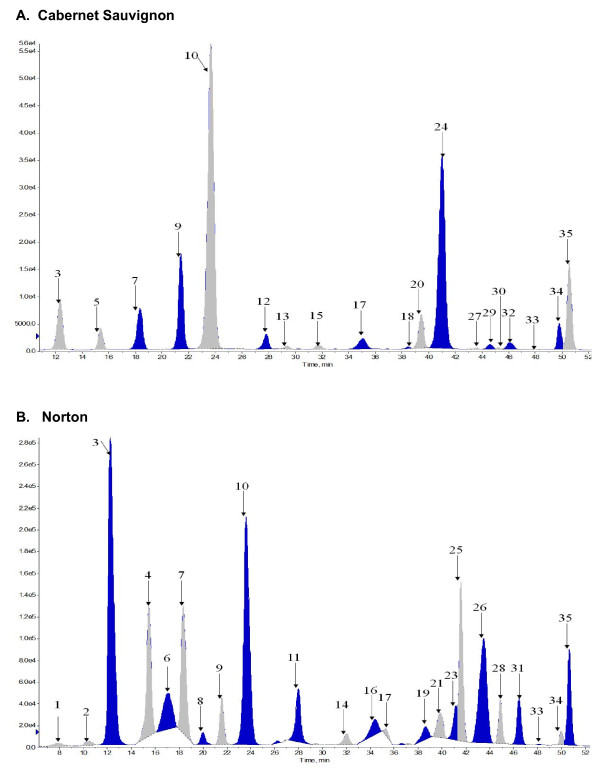
**HPLC chromatograms of anthocyanin compounds in the berry skin of *V. vinifera *'Cabernet Sauvignon' (A) and *V. aestivalis *'Norton' (B) at harvest ripe (stage 38)**. More anthocyanin compounds were found in Norton berry skin than in Cabernet Sauvignon. The identified compounds from each profile are listed in Table 3. The HPLC conditions are described in Materials and Methods.

The differences detected in the accumulation of cyanidin-, peonidin-, delphinidin-, petunidin- and malvidin derivatives prompted us to compare anthocyanin profiles of ripe Norton and Cabernet Sauvignon berry skin in detail. We used liquid chromatography-tandem mass spectrometry (LC-TIS/MS/MS) to identify the anthocyanin compounds. Thirty five different anthocyanins were identified in the two grape varieties (Table 3 and Figure 7). Eight of the 35 compounds were common to both varieties; sixteen of them were detected only in Norton. Norton-specific compounds include those previously described 3'-5' diglucoside derivatives as well as a number of sophoriside-glucosides and p-coumaryl-glucosides. Rutinoside derivatives appear to be unique to Cabernet Sauvignon. Cabernet Sauvignon had a single diglucoside anthocyanin, namely petunidin 3',7'-diglucoside (Table 3).

**Table 3:** Anthocyanins detected in the berry skin of ripe Norton and Cabernet Sauvignon grapes.

Anthocyanins	**Compound ID**^**A**^		Molecular ion: Product ion
		
	Norton	Cabernet Sauvignon	
**Compound detected in both varieties**			
Delphinidin 3-glucoside	3	3	465: 303
Cyanidin 3-glucoside^B^		5	449
Petunidin 3-glucoside	7	7	479: 317
Peonidin 3-glucoside	9	9	463: 301
Malvidin 3-glucoside	10	10	493: 331
Petunidin 3-(6''-acetylglucoside)	17	17	521: 317
new pigment B	33	33	677
Peonidin 3-*O-cis-p-*coumarylglucoside	34	34	609
Malvidin 3-*O-trans-p*-coumarylglucoside	35	35	639
**Compound detected only in Norton**			
Delphinidin 3,5-diglucoside	1		627: 465, 303
Cyanidin 3,5-diglucoside	2		611: 449, 287
Peonidin 3,5-diglucoside	4		625: 463, 301
Malvidin 3,5-diglucoside	6		655: 493, 331
Delphinidin 3-arabinoside	8		435: 303
Malvidin 3-(6''-acetylglucoside)-5-glucoside	11		697: 535, 493, 331
Cyanidin 3-(acetylglucoside)	14		491: 287
Delphinidin-3-(6-*O-p-*coumarylglucoside)-5-glucoside	16		773: 611, 465, 303
Malvidin 3-sophoroside-5-glucoside	19		817: 655, 493, 331
Petunidin 3-(6''-p-coumarylglucoside)-5-glucoside	21		787: 625, 479, 317
Petunidin 3-sophoroside	22		641
Malvidin 3-(6''-acetylglucoside)	23		535: 331
Delphinidin 3-*O*-*p*-coumarylglucoside	25		611: 303
Malvidin 3-(6-*O-p-*coumarylglucoside)-5-glucoside	26		801: 639, 493, 331
Cyanidin 3-*O-p*-coumarylglucoside	28		595: 287
Petunidin 3-*O-trans-p*-coumarylglucoside	31		625: 317
**Compound detected only in Cabernet Sauvignon**			
Delphinidin 3-(6''-acetylglucoside)		12	507: 303
Petunidin 3,7-di-glucoside		13	641
Delphinidin 3-O-beta-D-glucopyranoside		15	465
New pigment A		18	573: 369
Peonidin 3-(6''-acetylglucoside)		20	505: 301
Cyanidin 3-(3''-malonylglucoside)		24	535
Petunidin 3-rutinoside		27	625: 301, 317
Malvidin 3-gentiobiside		29	655: 331
Peonidin 3-rutinoside		30	609: 301
Malvidin 3-rutinoside		32	639: 331

^A ^The compound ID corresponds to the labels of the liquid chromatography peaks in Figure 7. ^B ^compound cyanidin 3-glucoside was detected in both varieties by HPLC with Agilent instrument as shown in Figure 6

At 127 DAB, anthocyanin diglucosides contribute 59% of the total anthocyanins in Norton berry skin. The major anthocyanins are malvidin derivatives that contribute 49% (5.73 mg/g FW) to total anthocyanins, followed by delphinidin (17%), petunidin (11%), peonidin (12%), and cyanidin (7%). In Cabernet Sauvignon, the main anthocyanin component is malvidin-3'-glucoside, which contributes 67% (1.82 mg/g FW) to the total anthocyanin amount at 112 DAB, followed by peonidin (14%), delphinidin (10%), petunidin (6.8%) and cyanidin (2%). Overall, in harvest-ripe berries, the total anthocyanin content in Norton berry skin (11.59 mg/g FW) is considerably higher than in Cabernet Sauvignon berry skin (2.70 mg/g FW).

### Expression profiles of key genes and accumulation of anthocyanins and PAs display a good correlation in Norton berry skin

**Figure 8:**
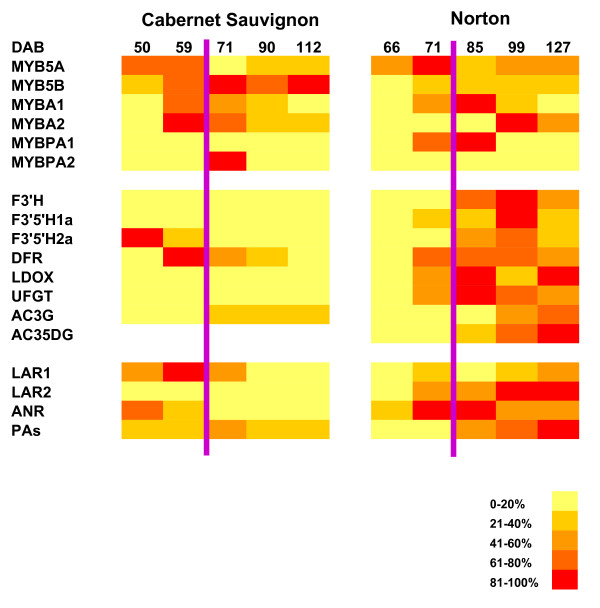
**A concise representation of qPCR and HPLC data for visualizing the coordination of transcriptional regulation of the genes and the total amounts of anthocyanins and proanthocyanidins in Cabernet Sauvignon and Norton berry skin**. DAB, days after bloom; AC3G, total amounts of anthocyanins-3-O-glucoside; AC35DG, total amounts of anthocyanins-3,5-di-O-glucoside; PAs, total amounts of proanthocyanidins. Abbreviations of the genes are the same as in Figure 2. Purple bar indicates the véraison phase. The heatmaps were generated by dividing the transcript abundance for each gene as in Figure 3 and 4, and the concentration of total anthocyanins and PAs as in Figure 5 and 6 into 5 percentiles of the highest level. The color legend represents the abundance of transcripts and metabolites in percentage range of the highest level for each gene and for total AC3G, AC35DG, and PAs.

A concise summary of coordinated transcription of key genes and biosynthesis of anthocyanins and PAs in the developing berry skin is presented in Figure 8. Transcript levels of *F3'H *and *F3'5'H1a/2a *peaked at 99 DAB and were higher in Norton than in Cabernet Sauvignon (Figure 3). We speculate that more flavonoid precursors (dihydroflavonols) are produced that are converted to anthocyanins and PAs in Norton than in Cabernet Sauvignon. This speculation is supported by the patterns and levels of accumulation of anthocyanins and PAs during berry development of the two varieties (Figure 5 and 6). One *DFR *gene (GSVIVT00014584001) displayed enhanced expression at the onset of véraison and remained at steady levels in Norton berry skin post-véraison, as measured by both qPCR (Figure 4) and microarray analyses (cluster 1, Figure 1 and Table 2). The constantly high mRNA levels of this *DFR *gene likely result in consistent production of leucoanthocyanidins that are substrates for LAR. Transcripts of *LAR1 *and *LAR2 *increased gradually after véraison (Figure 4), concurrently with catechin accumulation (Figure 5).

LDOX catalyzes the last two steps of anthocyanin synthesis (Figure 2B). The transcriptional profile of one LDOX gene (GSVIVT00001063001) showed increasing levels until 85 DAB, declining at 99 DAB, and increasing to the final stage in Norton berry skin, as observed in both microarray (cluster 13, Figure 1 and Table 2) and qPCR analyses (Figure 3). Transcripts of *LDOX *are more abundant in Norton than in Cabernet Sauvignon throughout the ripening phase (Figure 3). The highest transcript levels of one *ANR *gene (GSVIVT00005344001) at the onset of véraison declined gradually during ripening (Figure 1, cluster 10 and Figure 3), which is in agreement with the pattern of epicatechin accumulation (Figure 5).

UFGT catalyzes the last step in the anthocyanin biosynthesis pathway (Figure 2B). MYBA1 and MYBA2 regulate the transcription of *UFGT *[21,67,68]. Transcript levels of *MYBA1*/*A2 *peaked at véraison (59 DAB) in Cabernet Sauvignon, and post véraison at 85 and 99 DAB in Norton. Correspondingly, transcripts of one *UFGT *gene (GSVIVT00014047001) reached maximum levels at 85 DAB in Norton, but were found to be at significantly lower levels in Cabernet Sauvignon. The synchronized expression patterns of *MYBA1*/*A2 *and *UFGT *in both varieties suggest a close correlation between the transcription factors and their target genes. The higher transcript levels of *UFGT *in Norton than in Cabernet Sauvignon post-véraison (Figure 3) correlate remarkably well with the higher content of total anthocyanins in Norton berry skin at harvest (Figure 6).

## Conclusions

In summary, developmentally regulated resistance of Norton ripening berry to pathogens likely is a result of the steady increase of transcript abundance of *R *genes, *PR-1*, stilbene synthase genes, and genes of the phenylpropanoid pathway along the berry skin development. The expression patterns of six MYB transcription factor genes and their target structural genes in the anthocyanin and PA biosynthesis pathways correlate highly with the accumulation patterns of three PA compounds and five classes of anthocyanins. MYBPA1 and MYB5A may play more significant roles in the regulation of the flavonoid biosynthesis pathway in Norton than in Cabernet Sauvignon, whereas MYBPA2 and MYB5B appear to be more important in Cabernet Sauvignon than in Norton. The concomitant modulation of anthocyanin biosynthesis at the transcriptional level leads to more abundant production of anthocyanins in Norton berry skin in comparison with Cabernet Sauvignon berry skin.

## Methods

### Collection of berry skin

Berries from *V. vinifera *'Cabernet Sauvignon' and *V. aestivalis *'Norton' were collected at six developmental stages during the 2008 growing season from vines grown in a vineyard in the Missouri State Fruit Experiment Station, Mountain Grove, Missouri, USA, according to the phenological developmental stages defined by Coombe [69]. The berries were sampled at the following stages: 31 (pea-sized), 33 (still hard), 34 (softening), 35 (véraison), followed by 36, 37 and 38 (harvest ripe). Berry skin was separated from pulp, and pulp tissues were further removed by rubbing the internal side of the skin against filter paper. The cleaned skin tissues were immediately frozen in liquid nitrogen and stored at -80°C.

### RNA extraction and cDNA synthesis

Total RNA was extracted from the skin tissue according to the procedure of Reid et al. [70], using a CTAB-spermidine extraction buffer. Total RNA was treated with 1 unit of DNase I (Ambion, Austin, Texas, USA) for 30 minutes at 37°C and purified using RNeasy MinElute Cleanup kit (Qiagen, Valencia, California, USA). RNA quantity and quality were assessed by Agilent 2100 Bioanalyzer (Agilent Technologies, Santa Clara, California, USA). For cDNA synthesis, two μg of total RNA was reverse transcribed with oligo-dT in a 20 μl reaction mixture using the MultiScribe reverse transcriptase (Applied Biosystems, Branchburg, New Jersey, USA) according to the manufacturer's instructions.

### Microarray hybridization and data processing

Array hybridization was performed at the DNA Core Facility, University of Missouri (Columbia, Missouri). A total of 0.5 μg of total RNA was used to make the biotin-labeled antisense RNA (aRNA) target using the MessageAmp™ Premier RNA amplification kit (Ambion, Austin, Texas) following the manufacturer's protocol. Briefly, total RNA was reverse transcribed to first strand cDNA with an oligo(dT) primer bearing a 5'-T7 promoter using ArrayScript reverse transcriptase. First strand cDNA then underwent second-strand synthesis to convert it into double stranded cDNA as a template for *in vitro *transcription. The biotin-labeled aRNA was synthesized using T7 RNA transcriptase with biotin-NTP mix. After purification, the aRNA was fragmented in 1× fragmentation buffer at 94°C for 35 min. One hundred and thirty μL of hybridization solution containing 50 ng/μl of fragmented aRNA was hybridized to the Affymetrix GRAPEGEN GeneChip (Affymetrix, Santa Clara, California) at 45°C for 20 hrs. After hybridization, the chips were washed and stained with R-phycoerythrin-streptavidin in an Affymetrix fluidics station 450 using fluidics protocol Midi_euk2v3-450. The image data were acquired by Affymetrix GeneChip scanner 3000 and Affymetrix GCOS software.

### Annotation of probe sets and clustering

The Affymetrix microarray (GRAPEGEN GeneChip) used in this analysis included probe sets for 23,096 unigenes [30]. The intensity data of all genes on the microarray were analyzed by ANOVA with the Benjamini-Hochberg False Discovery Rate Multiple Test Correction method and applying a *p*-value of 0.001. The resulting data set was further reduced by applying a cut-off fold change of 2 or greater, which led to a final set of 3,352 significantly changed probe sets.

To annotate the putative function of the 3,352 probe sets that exhibited significant expression changes during berry development, the FASTA sequences were BLAT-searched against the 8× genomic sequences of *V. vinifera *PN40024 (http://www.genoscope.cns.fr/externe/GenomeBrowser/Vitis/) by using each FASTA sequence as query to acquire a Genoscope ID number. If no Genoscope ID was found for the query sequence, a Tentative Consensus (TC) ID was retrieved from VVGI5 database (http://compbio.dfci.harvard.edu/tgi/cgi-bin/tgi/gimain.pl?gudb=grape). The latest annotations for all Genoscope IDs and relational Network IDs, InterPro domain IDs, Gene Ontology IDs, UniProtIDs, TCs and functions have been published (Table S1, [71]), and were used as the reference for functional category and annotation. The original annotations by the GeneChip manufacturing group were also cross-referenced for verification.

More than one sequence was annotated with the identical Genoscope or DFCI ID in 401 cases, which brought the total number of unigenes down to 2,760. All genes with multiple annotations and four sequences for which neither a Genoscope annotation nor a DFCI match were found were removed from the data set, resulting in 2,359 unigenes.

The expression profiles of the 2,359 unigenes were clustered using the *k*-means method with Pearson's correlation as distance. They were grouped into 20 clusters after evaluation of the Figure of Merit (FOM) graph in the Multiple Experiment Viewer version 4.4 software package.

### Quantitative real-time PCR (qPCR)

Transcript levels in grape skin were measured by quantitative real-time PCR, using SYBR Green in the MX3005P system (Stratagene) following the manufacturer's manual. The reaction mixture (20 μl, in triplicate) contained 0.5 μl 1:10 diluted cDNA as a template and 20 pmole each of the forward and reverse primers specific to each gene. The primers were designed from the 3'-UTR region to avoid any unspecific amplification. Thermal cycling conditions were as follows: 95°C for 10 min, 65 cycles of 95°C for 15 sec, 60°C for 30 sec and 1 cycle of 95°C for 1 min, 60°C for 30 sec and 95°C for 30 sec. The annealing temperature (60°C) was determined computationally when designing the primer. The melt curves for the products of these assays produced a single peak, indicating that a single gene had been amplified. The specificity of each primer pair was also checked by gel electrophoresis and by sequencing the PCR products and comparing them with the sequence of the target gene. PCR efficiency (*E*) was calculated from the exponential phase of each individual amplification plot and the equation (1 + *E*) = 10^slope ^based on a previous method [72]. Expression levels of genes of interest (GOI) were normalized to that of *ACTIN *by dividing the C_T _value of GOI by the C_T _value of *ACTIN*. Gene expression was expressed as mean and standard error calculated based on three biological replicates.

### Reverse phase HPLC analysis of anthocyanins and proanthocyanidins

For anthocyanin extraction, frozen berry skin tissue was ground in liquid nitrogen, and 500 mg of the ground tissue was extracted with 5 mL acidified methanol (60% (V/V) methanol containing 0.1% (w/V) ascorbic acid) for 24 hours on a shaker in the dark at room temperature. The extracts were centrifuged twice at 16,100 g for 10 minutes. The final supernatants were kept in the dark and refrigerated until analysis; two samples were prepared from each biological replicate.

For proanthocyanidin extraction, frozen seeds or frozen berry skin were ground in liquid nitrogen, and 500 mg of ground tissue was used for extraction in 5 ml extraction buffer (70% [V/V] acetone containing 0.1% [w/V] ascorbic acid) for 24 hr at room temperature on a rotating shaker in darkness. The water phase was separated from the acetone phase by adding sodium chloride to saturation. After removal of the acetone phase, the water phase was extracted with additional sodium chloride-saturated 100% acetone, and the resulting acetone phase was combined with the first acetone phase. The samples were dried under a stream of nitrogen, the pellet re-dissolved in 750 μL of 60% methanol acidified with 0.1% ascorbic acid, centrifuged at 16,100 g for 10 minutes, and the final supernatant kept in darkness and under refrigeration until analysis; two samples were prepared from each biological replicate.

Anthocyanin and proanthocyanidin content and composition were determined by reverse-phase HPLC using an HP1100 series (Agilent) Chemstation, with a Zorbax Eclipse XDB-C18 (80 Angstrom, 4.6 × 150 mm, particle size 3 μm) column with a guard column. The binary solvent system of solvent A (acetonitrile (HPLC grade, EMD Chemicals, USA) and Solvent B (2% phosphoric acid [(HPLC grade, Sigma Aldrich), V/V Millipore water] was used for both the anthocyanin and the proanthocyanidin analyses. The gradient used for anthocyanin separation was as follows: acetonitrile 6% for 3 min; 8% for 24.50 min; 10% for 22.50 min; 18% for 23.50 min; 90% for 4.5 min; and 8% for 7 min; with a flow rate of 0.8 mL/min for 36 minutes, then 0.6 mL/min for 49 min. The gradient used for proanthocyanidin separation was as follows: acetonitrile 8% for 5 min, 12% for 12 min, 20% for 10 min, 25% for 6 min, 50% for 2 min, 80% for 7 min, 8% for 5 min; with a flow rate of 0.5 mL/min. In each case, the column was maintained at 40°C and the diode array detector was used to record absorption at 280 nm, 335 nm and 520 nm. Malvidin-3-glucoside chloride, catechin hydrate, epicatechin, epicatechin gallate, epigallocatechin, epigallocatechin gallate and proanthocyanidin B2 (all HPLC grade, Sigma-Aldrich) were used to create standard absorption curves. All anthocyanins were expressed as malvidin glucoside equivalents based on the peak areas recorded at 520 nm with a molecular weight correction factor applied. The peak areas recorded at 280 nm in conjunction with the respective standard absorption curves were used to express the proanthocyanidins as mg per gram of fresh weight.

### LC-TIS/MS/MS analysis of anthocyanins

Anthocyanins were extracted by following the protocol for extracting proanthocyanidins as described in the previous section. All samples were analyzed using a 4000 QTRAP LC-TIS-MS-MS system (Applied Biosystems, Forest City, CA) by monitoring the enhanced product ion (EPI) and multiple reaction monitoring (MRM) in the positive ionization mode. Separation of (10 μL) samples was achieved by using a Gemini-NX C18 HPLC column (Phenomenex, 5 μm, 150 mm × 2 mm) combined with a C18 guard column (Phenomenex, 4 mm × 2 mm). The mobile phase flow was set to 0.45 mL/min with binary gradient elution, using solvent A (aqueous 5% formic acid solution) and B (95% CH_3_CN, 5% formic acid). The gradient was as follows: 0-3 min, 5% B; 3-15 min, 5-9% B; 15-27 min, 9-13.5% B; 27-32 min, 13.5% B, 32-42 min, 13.5-18.5% B; 42-44 min, 18.5% B; 44-51 min, 18.5-22.5% B; 51-55 min, 22.5-30% B; 55-56 min, 30-40% B; 56-60 min, 40-70%; 60-60.1 min, 70-100% B; 60.1-70 min, 100% B; 70.0-70.1 min, 100-5% B; 70.1-80 min, 5% B. The elution of anthocyanins was monitored at 520 nm. The following TIS source parameters were used: CUR 30 eV, CAD high, IS 5500, TEM 550°C, DP 40 eV, CE 10 eV. The mass scan range was 50 to 1000. For anthocyanin quantification, five anthocyanin standards (Chloride salt of delphinidin (Sigma, MO), cyanidin (Chromadex, CA), petunidin (Chromadex, CA), peonidin (Chromadex, CA) and malvidin (Chromadex, CA) were used to create a calibration curve for each anthocyanin. All calibration curves were linear, with R^2^≥0.998.

## Abbreviations

PAL: phenylalanine ammonia-lyase; C4H: cinnamate 4-hydroxylase; 4CL: 4-coumarate-CoA ligase; CAD: cinnamyl alcohol dehydrogenase; CCoAOMT: caffeoyl-CoA 3'-O-methyltransferase; COMT: caffeic acid O-methyltransferase; CCR: cinnamoyl-CoA reductase; F5H: ferulate-5'-hydroxylase; STS: stilbene synthase; CHS: chalcone synthase; CHI: chalcone isomerase; UFGT: UDP-glucose:flavonoid-3-O-glucosyltransferase; F3H: flavanone 3-hydroxylase; F3'H: flavonoid-3'-O-hydroxylase; F3'5'H: flavonoid-3',5'-hydroxylase; DFR: dihydroflavonol-4-reductase; LDOX: leucoanthocyanidin dioxygenase; ANR: anthocyanidin reductase; LAR: leucoanthocyanidin reductase; GST: glutathione S-transferase; OMT: O-methyltransferase; PA: proanthocyanidin.

## Authors' contributions

MBA extracted total RNA, analyzed RNA quality, performed qPCR, made graphs and assisted in annotating genes, analyzing data and drafting manuscript. SH collected samples, performed chemical analysis of berries, clustering of microarray data and statistical analysis of qPCR results, conducted HPLC, and assisted in annotation of genes. SC established and optimized HPLC conditions for analyzing anthocyanins and PAs. YW and OY performed LC-TIS/MS/MS analysis. LGK conceived, designed and supervised the experiments, collected samples and contributed to manuscript writing. WQ conceived the comparative study between the two grape varieties; supervised qPCR assays, annotation and clustering of genes, and drafted and finalized the manuscript. All authors were involved in editing and revising the manuscript.

## Supplementary Material

Additional file 1 **Principal Component Analysis (PCA) of the eighteen set of microarray hybridization data**. Six stages (Stage 33 to 38) are denoted by different colors. Filled rectangle, rectangle, and filled circle represent three biological replicates. Click here for file

Additional file 2 **Hierarchical cluster analyses of the eighteen sets of data for assessing the quality of the data**. Click here for file

Additional file 3 **Pearson correlation coefficient analysis of the eighteen set of data in pair-wise**. Click here for file

Additional file 4 **A list of 15,823 probe sets that exhibited significant variations along six stages (at p-value ≤ 0.001)**. This list of probe sets was generated by conducting ANOVA on error-weighted intensity experiment definitions (EDs). Sequence description: Brief narrative description of gene annotation; Grand average: the average value of each probe set intensity across all factor levels in the ANOVA, and this average was computed after error-weighting; The Pooled Variance: the within mean square for each gene-analysis level item across all factor levels; Group p-value: the probability that the null hypothesis--that expression levels or differential expression ratio levels are not significantly different across factor levels--is not true. A low p-value indicates high confidence that the gene's expression level or ratio level is significantly different across the groups defined in the ANOVA. Click here for file

Additional file 5 **A list of 3,352 probe sets that exhibited significant variations along six stages (at p-value ≤ 0.001) with a ratio of more than 2**. The legends of each column are the same as in Additional file 4. This list of probe sets was determined by conducting error-weighted ANOVA. Click here for file

Additional file 6 **Cluster analysis of the transcript abundance of the differentially expressed 2,359 unigenes across six developmental berry skin stages**. Click here for file

Additional file 7 **GenBank accession number, Genoscope number, TC number, GeneChip ID number, primer sequences, expected size and sequences of amplified DNA fragments of the genes that were analyzed in the berry skin of Norton and Cabernet Sauvignon by the quantitative real-time PCR (qPCR)**. The qPCR-amplified DNA fragments were sequenced to verify the identity of each amplicon. Correlation coefficient analysis of the transcript levels between qPCR and microarray was also included. Click here for file
