# Supplementary figures and images for: Berry skin development in Norton grape: Distinct patterns of transcriptional regulation and flavonoid biosynthesis
Source: BMC Plant Biol. 2011 Jan 10;11:7. doi: 10.1186/1471-2229-11-7 (PMC3025947; doi:10.1186/1471-2229-11-7)

## Slide 1
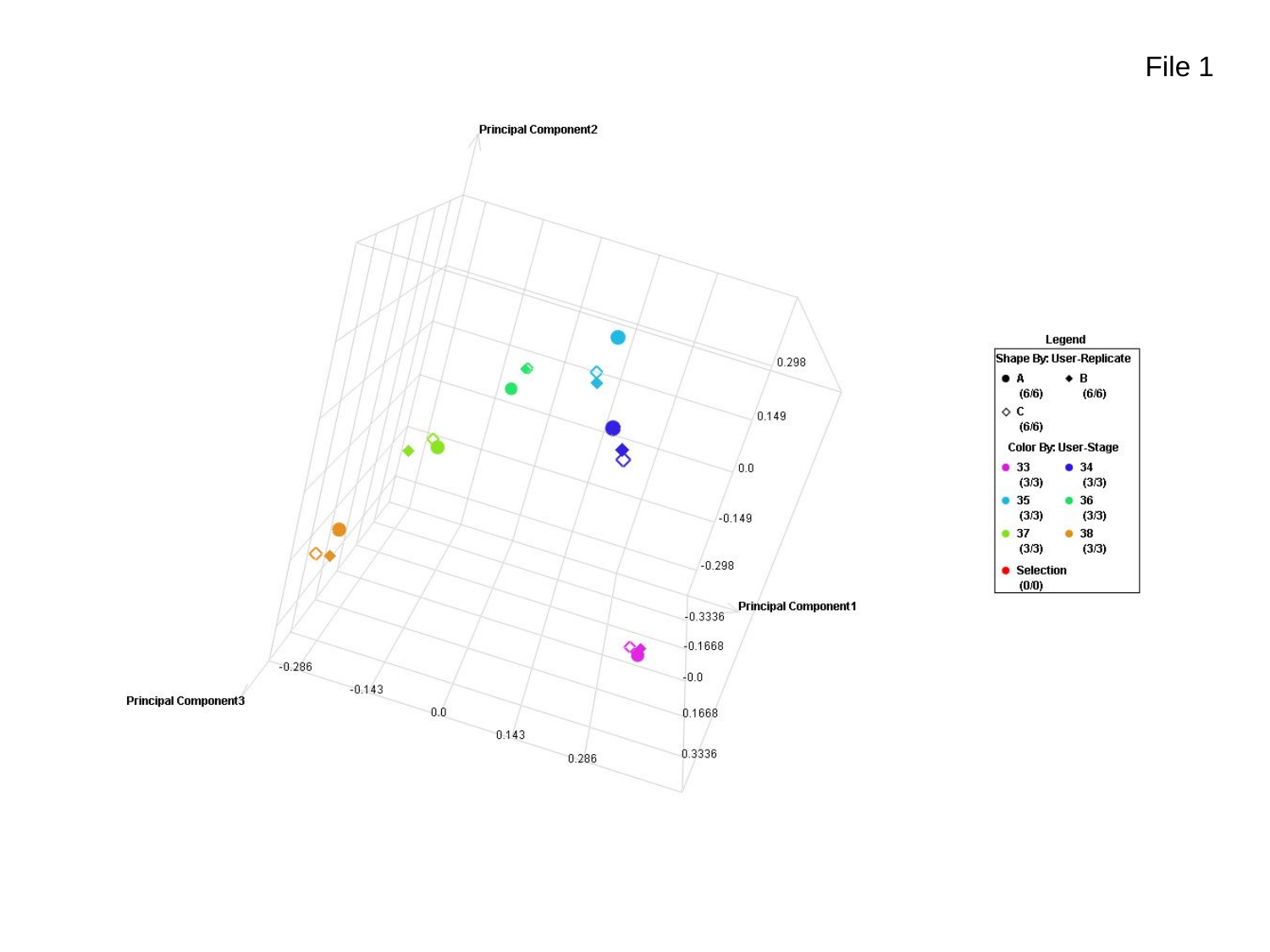

File 1

Supplement: Additional file 1 — Principal Component Analysis (PCA) of the eighteen set of microarray hybridization data. Six stages (Stage 33 to 38) are denoted by different colors. Filled rectangle, rectangle, and filled circle represent three biological replicates. [file 1471-2229-11-7-S1.PPTX]

## Slide 1
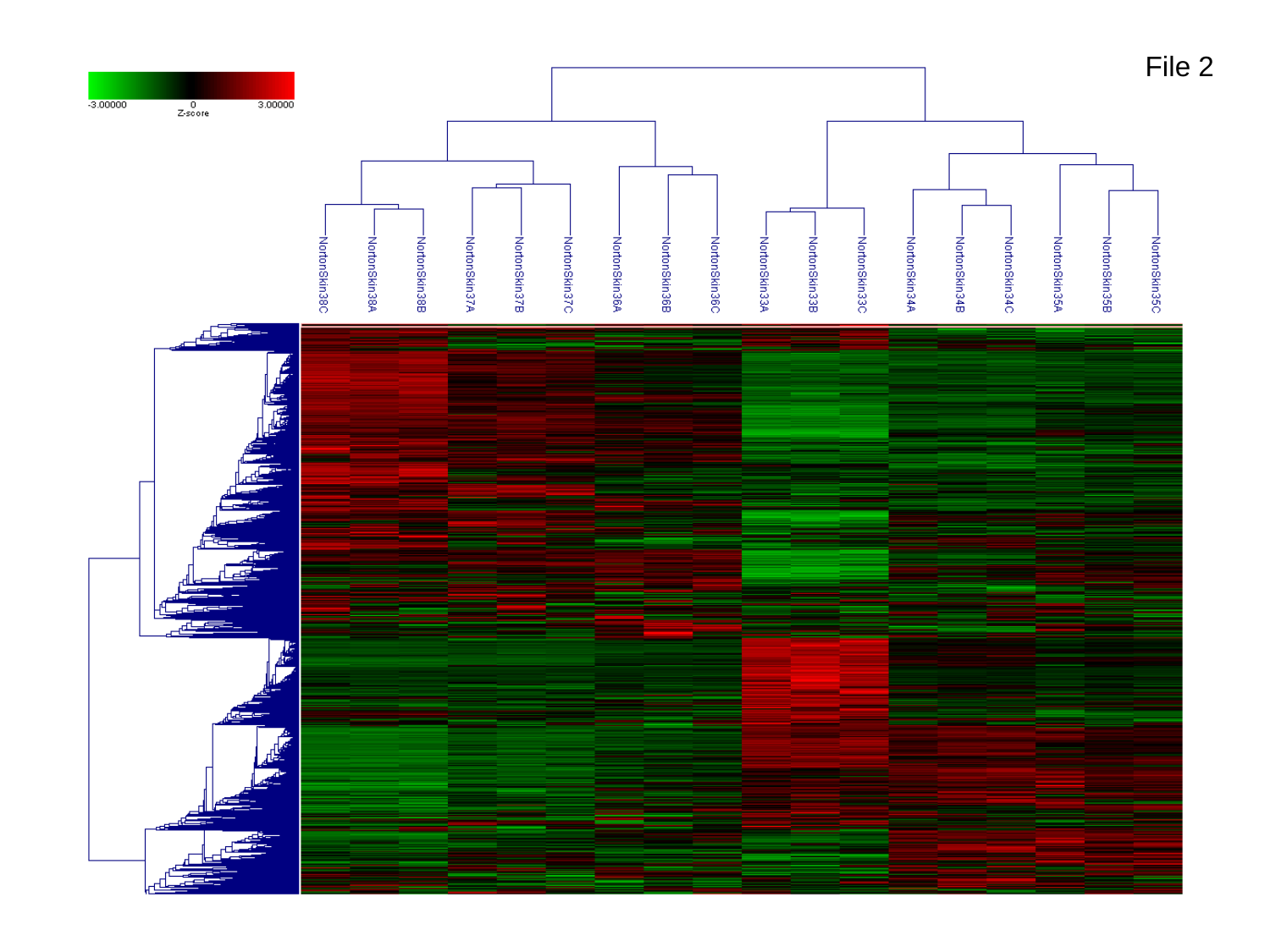

File 2

Supplement: Additional file 2 — Hierarchical cluster analyses of the eighteen sets of data for assessing the quality of the data. [file 1471-2229-11-7-S2.PPTX]
